# Adenosine: a selfish-immunity signal?

**DOI:** 10.18632/oncotarget.4685

**Published:** 2015-06-29

**Authors:** Tomas Dolezal

**Affiliations:** University of South Bohemia, Ceske Budejovice, Czech Republic

**Keywords:** Immunology and Microbiology Section, Immune response, Immunity

It is long known that immune response is energydemanding. It has become clear in recent years [1] that pretty much any immune cell in our body undergoes, upon its activation, a metabolic shift resembling the Warburg effect originally described for cancer cells. Immune cells increase glucose consumption and produce a significant portion of ATP by glycolysis ending with lactate even under oxygenated conditions; increased glycolysis is required for the generation of intermediate metabolites associated with the activation of the immune cell.

Increased energy consumption by immune cells requires a metabolic adaptation of the whole organism. During trauma or infection, the organism vitally depends on the immune system, which is therefore privileged in energy/nutrient allocation. According to Rainer Straub [2], insulin resistance caused by pro-inflammatory cytokines is a physiological way of the immune system to usurp energy/nutrients during acute stress from the rest of the organism because immune cells themselves do not become insulin resistant. Such selfish behavior of the immune system may be crucial for an effective immune response.

We have recently demonstrated a selfish behavior of the immune system during defense of Drosophila larva against parasitoid wasp infection [3]. The wasp injects its egg into the larva and that activates a production of specialized immune cells called lamellocytes, which encapsulate and destroy the parasitoid egg. Production of lamellocytes is associated with increased glycolysis and glucose consumption by precursors of these cells. We demonstrated that a systemic metabolic switch, which included a suppression of development and energy storage, was required for the rapid production of lamellocytes and thus for the effective immune response. We further showed that lamellocytes precursors released adenosine that suppressed the consumption of glucose by non-immune tissues and thus slowed down the host development. When we blocked adenosine signaling or its release from immune cells, the development proceeded with normal speed but the resistance against parasitoid dropped, demonstrating a trade-off between development and the immune response. In our experimental system, immune cells use adenosine as a selfish signal to usurp energy from the rest of the organism, which is a vital strategy during infection.

Extracellular adenosine can be produced in extracellular space from ATP, which for example leaks out from damaged tissues. Alternatively, when demand for ATP exceeds supply in a cell, the decreasing ATP level results in an increased level of AMP that can either activate AMPK and thus can suppress energy consuming processes within the cell or AMP can be converted to adenosine by cytosolic 5′-nucleotidase [4]; adenosine is then released to extracellular space by equilibrative nucleoside transporters where it can inform other tissues about the metabolic stress. The conversion of AMP to adenosine, instead of activating AMPK, would make more sense for activated immune cells, which need to obtain more energy; it remains to be tested if this was the origin of adenosine whose effects on systemic metabolism were observed in our work [3]. Extracellular adenosine could thus represent another type of selfish immune system signal - unlike proinflammatory cytokines, which would rather measure the robustness of the immune system activation (e.g. how many immune cells have been activated), adenosine would measure the actual energy needs of the immune cells and the actual tissue damage (ATP leakage).

Can adenosine play a similar role in higher organisms including humans? Adenosine is produced, for example, by activated neutrophils and its systemic level is increased during sepsis. Adenosine generally suppresses energy-consuming processes; this can be observed both at the cellular level, e.g. inhibiting cell growth, and at the systemic level. The systemic suppression effects of adenosine are observed in torpor/hibernation and are important for anoxia-tolerant organisms. Adenosine is known to suppress neuronal firing and to induce sleep; caffeine is the most famous adenosine receptor antagonist. Increased plasma levels of adenosine were associated with chronic fatigue syndrome and adenosine was shown to mediate an exercise-induced fatigue [5]. Fatigue is a hallmark of sickness and thus it is tempting to speculate that adenosine may cause fatigue in proportion to tissue damage and the energy needs of immune cells. Fatigue and suppressing the overall activity of the organism could form, together with insulin resistance, a complex program to conserve energy for the immune system.

**Figure 1:**
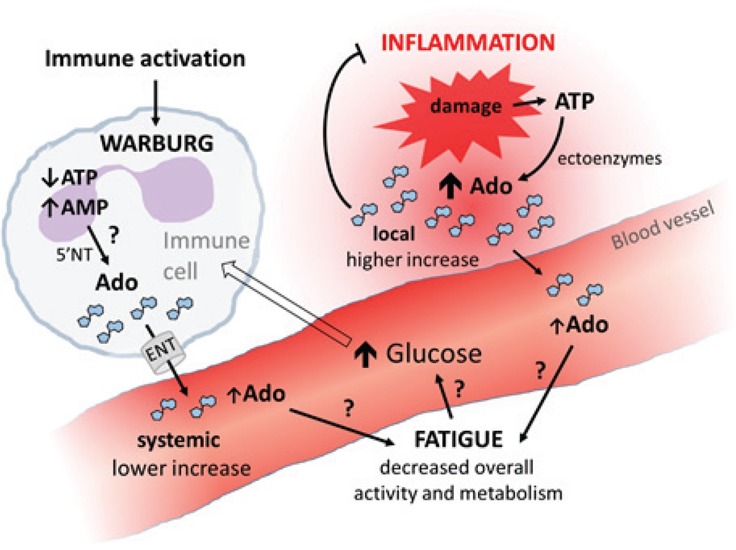
Speculative role of adenosine in metabolic regulation during immune response Ado…adenosine, 5′NT…cytosolic 5′-nucleotidase, ENT… equilibrative nucleoside transporter; blue polygons represent molecules of adenosine.

How would this role of adenosine go together with its well-established anti-inflammatory role in the mammalian immune system [6]? The key might be to distinguish local and systemic effects, effects of different levels of adenosine and timing (Figure 1). Low circulating levels of adenosine (though increased above the basal level) might have little effect on immune cells (or rather a stimulatory effect) but may have systemic suppressive effects influencing energy distribution within the organism. High levels of adenosine, generated by damaged tissues in sites with excessive inflammation, have anti-inflammatory effects on immune cells at that site; adenosine leaking into circulation from inflamed sites may further contribute to energy regulation at the systemic level.

It would be interesting to test if adenosine, produced during immune response, influences the metabolism of non-immune tissues, activity and wakefulness, regulating thus the energy allocation in higher organisms. How important would such metabolic regulation be for the effectivity of immune response? It might be difficult to study these complex roles of adenosine but purinergic signaling is an important target for new drugs, for example to treat inflammatory diseases [6], and thus it is important to understand the various roles of adenosine.
